# Machine Learning-Assisted Improved Anomaly Detection for Structural Health Monitoring

**DOI:** 10.3390/s23073365

**Published:** 2023-03-23

**Authors:** Shreyas Samudra, Mohamed Barbosh, Ayan Sadhu

**Affiliations:** Department of Civil and Environmental Engineering, Western University, London, ON N6A 3K7, Canada

**Keywords:** anomaly detection, structural health monitoring, vibration data, machine learning, decision tree, random forest

## Abstract

The importance of civil engineering infrastructure in modern societies has increased lately due to the growth of the global economy. It forges global supply chains facilitating enormous economic activity. The bridges usually form critical links in complex supply chain networks. Structural health monitoring (SHM) of these infrastructures is essential to reduce life-cycle costs, and determine their remaining life using advanced sensing techniques and data fusion methods. However, the data obtained from the SHM systems describing the health condition of the infrastructure systems may contain anomalies (i.e., distortion, drift, bias, outlier, noise etc.). An automated framework is required to accurately classify these anomalies and evaluate the current condition of these systems in a timely and cost-effective manner. In this paper, a recursive and interpretable decision tree framework is proposed to perform multiclass classification of acceleration data collected from a real-life bridge. The decision nodes of the decision tree are random forest classifiers that are invoked recursively after synthetically augmenting the training data before successive iterations until suitable classification performance is obtained. This machine-learning-based classification model evolved from a simplistic decision tree where statistical features are used to perform classification. The feature vectors defined for training the random forest classifiers are calculated using similar statistical features that are easy to interpret, enhancing the interpretability of the classifier models. The proposed framework could classify non-anomalous (i.e., normal) time-series of the test dataset with 98% accuracy.

## 1. Introduction

In recent decades, the economies of modern countries are interwoven, and their interdependence is growing, which has led to the creation of complex supply chain networks sustained by efficient civil engineering infrastructure facilitating enormous economic activities. Bridges are important infrastructures that are usually built to form critical links in complex supply chain networks [1]. Structural health monitoring (SHM) of bridges is critical to detect any abnormal behavior, provide timely maintenance, and increase their remaining-useful-life and reliability [2,3,4]. This necessitated the development of SHM techniques for the damage detection and condition assessment of civil structures [5]. SHM techniques are applied using non-contact and contact sensing techniques [3,6,7]. Contact sensing techniques use different types of sensors that are placed or attached to the monitored system, such as accelerometers, strain gauges, optical fibers, and acoustic emission. Out of them, accelerometer-based SHM techniques are the most cost effective and commonly used to monitor the overall condition of various systems [8,9,10]. However, accelerometers generate a large amount of time-series data that are often populated with anomalies reducing their effectiveness for long-term SHM. In this paper, an interpretable and computationally efficient machine learning (ML) framework is developed for accurate anomaly detection in SHM data.

Large-scale sensing-based SHM techniques used for real-time monitoring generate a large amount of time-series data. For instance, the SHM system used to monitor Sutong Bridge in China generated 2.5 TB of data collected from sensors in one year [11]. With such big data, it is impossible to analyze the data manually and detect anomalies, which makes system identification erroneous. Anomalies in the time series allude to physical realities influencing structural health, such as extreme climate conditions (e.g., strong wind, heavy rain, flood, very high or low temperature), the passage of overloaded vehicles, vehicle/ship collisions, and other instantaneous events [1,2,12]. However, all the anomalies cannot be attributed to degradation in structural health. Some anomalies can be caused due to sensor malfunction, calibration errors, noise, and transmission errors [2,12,13]. These anomalies can trigger false alarms. Several measures can be implemented on the hardware (i.e., sensor architecture) and software (i.e., sensor-data processing) to eliminate these anomalies. Hardware measures are the maximization of the use of wired transmission systems instead of wireless transmission systems, backup sensors (i.e., hardware redundancy), and self-validating sensors equipped with a computational unit capable of self-diagnosis, and self-calibration [14]. However, implementing these changes in sensor architecture is very expensive. Therefore, advanced data preprocessing techniques for anomaly detection are generally more popularly adopted [15].

The artificial intelligence (AI) methods can discover patterns in the time-series data without prior knowledge of the structural system [4]. The time-series data is subjected to time or frequency domain analysis to extract characteristic features representing the time series before applying supervised or unsupervised algorithms. The representative features can be obtained using statistical analysis or signal processing techniques such as coefficients of the Fourier transform, wavelet transform, Hilbert-Huang transform, and Shapelet transform [16]. Alternatively, the representative features can be automatically extracted using deep-learning (DL) algorithms, where the time-frequency information is fed as an input in the form of an image into a convolutional neural network (CNN) [3,11,15]. However, DL-based methods require high computational time and extensive hyperparameter tuning.

Some of the popular unsupervised ML algorithms are principal component analysis, k-means clustering, hierarchical clustering, and autoencoder [12]. However, feature-based clustering techniques that employ dimensionality reduction, such as self-organizing maps (SOM), are more popular [17]. However, the major drawback of unsupervised techniques is that their performance may deteriorate due to changes in the environmental conditions or operational conditions of the structure installed with the SHM system. In that case, expert intervention is needed for hyperparameter tuning to improve their performance for anomaly detection [18]. Unlike unsupervised ML methods, supervised algorithms can be broadly categorized into classification, regression, and ensemble learning. Popular classification algorithms used for SHM include the support vector machine (SVM), naive Bayes, Bayesian networks, and decision trees. Popular regression algorithms used for SHM include artificial neural networks (ANN), nonlinear regression, and support vector regression. Classification algorithms predict discrete classes, whereas regression algorithms predict a set of continuous values that can be used for classification by defining an appropriate threshold. Ensemble learning algorithms are constituted by several classifiers whose outputs are combined to predict the final output. The output of each classifier can be combined using bagging, boosting, or voting. Popular ensemble learning algorithms used for SHM include Adaboost, bagging, random forest, and [4,19].

The focus of this paper is to employ interpretable and computationally efficient machine learning (ML) techniques for the SHM of bridges using the anomaly in time-series data obtained from sensors. Some of the important works in this area are presented subsequently. For example, [12] developed a generative adversarial network and autoencoder-based unsupervised ML model to detect anomalies (i.e., binary classification) in acceleration time-series data of a long-span bridge in Jiangsu. In addition, [10] proposed a one-dimensional CNN and an autoencoder-based supervised DL model to detect anomalies (i.e., binary classification) in acceleration time-series data collected from a long-span steel box grinder suspension bridge in China. Moreover, [11,15] developed a CNN-based supervised DL model to detect anomalies (i.e., multiclass classification) in acceleration time-series data of a long-span cable bridge in China. Further, [20] developed an SVM-based supervised ML model to detect anomalies (i.e., binary classification) in simulated displacement time-series data corresponding to different locations of simple multicell beam structures that are used for bridge construction. In another study, [16] developed a shapelet transform-based supervised ML model using a random forest classifier to detect anomalies in acceleration time-series data of the long-span cable bridge in China (IPC SHM 2020). Additionally, [21] proposed a framework to detect anomalies in acceleration time-series data of the long-span cable bridge in China (IPC SHM 2020) by converting acceleration time-series data into grey scale time-series data and extracting multiview binary patterns to create feature vectors that are used to train random forest classifiers.

In this paper, a novel recursive decision tree-based supervised ML framework using a random forest classifier is proposed. This choice of structuring a decision tree with random forest classifiers is such as its decision nodes enables training and prediction with a low computational cost. Selecting other ML algorithms could have posed several challenges. For SVM, the selection of a good kernel is difficult, and the training time increases exponentially with the size of the dataset. K-means is prone to over-fitting for high-dimensional data, and Bayesian algorithms assume no interdependence of feature vectors, which may not always be true. However, for random-forest-based models, model interpretability is challenging. This was tackled by defining simple statistical features to train the ML models that are easy to interpret and computationally efficient to calculate. Moreover, the relative importance of the features was calculated using a predictor importance score, which can be used to further enhance the interpretability of the framework. While training ML models, unbalanced training datasets are synthetically augmented using synthetic minority oversampling technique (SMOTE) to improve classification performance. Thus, the proposed framework aims to overcome the challenges commonly observed in supervised ML frameworks, such as generalizability, interpretability, computational efficiency, and inadequate coverage of decision space because of unbalanced training data using a recursive decision tree framework with random forest classifiers as its decision nodes.

The paper is outlined as follows. First, a brief background of the supervised ML-based algorithm is provided, followed by a description of the dataset used to train, validate, and test the proposed ML-based algorithm. Then, a simplified method for anomaly classification employing a decision tree based on statistical features is proposed, and its extension using an ML algorithm is presented. Finally, the anomaly detection results are presented, and important conclusions are drawn.

## 2. Background

### 2.1. Anomalies in Time Series

**Figure 1:**
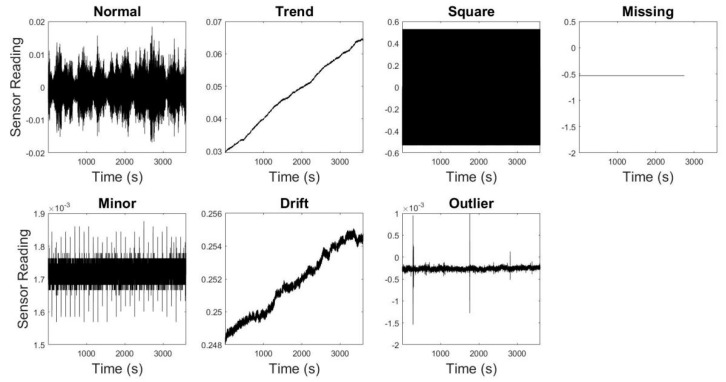
Typical time series of different anomalies.

Time series is a sequence of data points indexed with time (e.g., vibration data obtained from an SHM system). However, certain clusters of data points in the sequence may not follow the general patterns found in the measured data, which are commonly known as anomalies. A time series may contain several anomalies that can be attributed to different physical causes, such as sensor defects, noise, and others, depending on the physical reality of the data collection. This paper discusses the anomaly (i.e., trend, square, missing, minor, drift, and outlier) detection in acceleration time series obtained from a long-span cable bridge [22], as shown in Figure 1. Table 1 presents brief details of the anomalies considered in the data measured from the bridge. Besides the aforementioned anomalies, acceleration sensors attached to structures susceptible to damage can register other kinds of anomalous patterns, such as offset (i.e., response shows a sudden jump), gain (i.e., response starts increasing with time), precision degradation (i.e., response contains erratic patterns), and complete failure (i.e., response in frequency domain resembles white noise).

### 2.2. Synthetic Minority Oversampling Technique (SMOTE)

The datasets used to train ML models are often imbalanced (i.e., the number of data points belonging to different classes is not equal). These imbalances can lead to over-fitting or under-fitting, affecting the classification performance of the ML models by making false predictions (i.e., false positives or false negatives). This problem can be solved by synthetically augmenting the training data set corresponding to the classes of false positives and false negatives to train the ML models, commonly known as SMOTE [22]. It is the creation of artificial data from real data, and it ensures better coverage of the decision space of the ML algorithm corresponding to the minority class [23,24]. Therefore, SMOTE is used as the data augmentation tool for imbalanced data in this paper.

#1 *n*-dimensional vector space corresponding to *n* features of an ML model is defined where all the training data can be represented by feature vectors.
#2 Depending upon the amount of over-sampling required, *k* nearest neighbors (in terms of Euclidean distance) are randomly chosen.
#3 The difference between the feature vector (arbitrarily chosen from the set of *k* nearest neighbors) and its nearest neighbor is calculated.
#4 This difference is multiplied by a random number between 0 and 1, and a new feature vector is created at the corresponding location.

### 2.3. Minimum Redundancy and Maximum Relevance (MRMR) Algorithm

The feature selection for time-series data is crucial while building ML models for time-series classification. A set of features needs to be chosen to accurately represent a time-series consisting of a large amount of data. Selecting appropriate features can enhance the accuracy and computational efficiency of the classification algorithms. However, selecting highly correlated or irrelevant features may affect model performance because of redundancy, over-fitting, and poor accuracy of the classification algorithms. In this study, the maximum relevance and minimum redundancy (MRMR) feature selection method [25] is used to validate the relevance of features calculated to represent the SHM data measured from a real-life bridge.

The MRMR method calculates a set of features arranged in descending order of mutual correlation (or redundancy) and relevance toward the response variable or category. Redundancy and relevance are quantified using mutual information, which measures how much uncertainty of one variable can be reduced by measuring the other variable [26,27]. This algorithm is implemented to obtain predictor importance scores between 0 and 1, and predictor importance ranks of proposed features representing the time series. A higher predictor score indicates that the corresponding feature is more important, whereas a drop in the predictor importance score of a feature suggests a dip in the importance of the corresponding feature relative to a feature with a higher predictor importance score.

## 3. Data Analysis

### 3.1. Description of the Data and Adopted Performance Metrics

**Figure 2:**
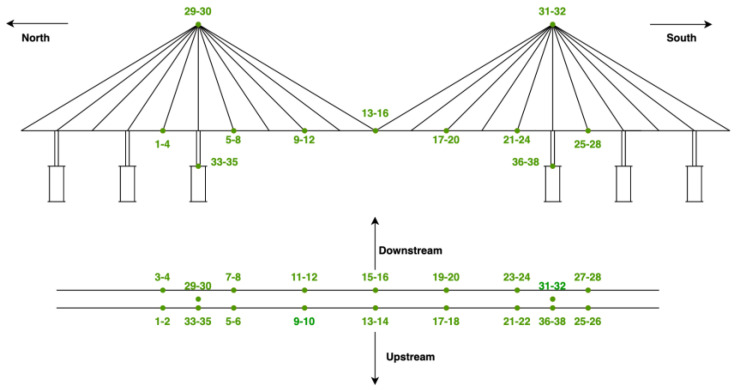
Schematic of the sensor location of the bridge [22].

The proposed method is validated using the dataset collected from a long-span cable-stayed bridge in China provided by the IPC-SHM community [22]. The dataset contains one month of acceleration data acquired using accelerometers placed at 38 locations on the bridge, as shown in Figure 2. The data is divided into one-hour-long time series (i.e., 38 locations × 31 days × 24 h = 28,272 time-series). The sampling frequency of the accelerometers is 20 Hz, resulting in 28,272 time-series × 3600 s × 20 Hz, i.e., 2 × 10^9^ data points. The 28,272 time-series responses are classified into seven different classes: the normal data and the six classes of anomalies (i.e., trend, square, missing, minor, drift, and outlier).

Table 2 shows the number of time-series belonging to different classes in the dataset. This dataset is divided into training, validation, and testing datasets. The training data is used to train a model capable of undertaking seven-class classification for anomaly detection. The validation data is used to enhance the classification performance of the trained models, and the testing data is used to evaluate the classification performance of the model using performance metrics, such as F1 score, accuracy, recall, and precision, as defined subsequently.

(1) $$Precision=\frac{TP}{TP+FP}$$

(2) $$Recall=\frac{TP}{TP+FN}$$

(3) $$Accuracy=\frac{TP+TN}{TP+TN+FP+FN}$$

(4) $$F1score=\frac{2\times Precision\times Recall}{Precision+Recall}$$

Here, TP is the number of true positives, TN is the number of true negatives, FP is the number of false positives, and FN is the number of false negatives.

### 3.2. Feature Engineering and Application of MRMR

To build a model based on a supervised ML algorithm, it is necessary to convert the raw data to certain features that represent the entire data set. This is accomplished by defining a set of 30 distinct features for each one-hour window of the time series after an extensive statistical analysis. The time-series data comprising 2 × 10^9^ data points compressed into 30 distinct features consumed only 0.022% (7 MB, i.e., compression ratio of 456) of the storage that was consumed by the original data. Data compression not only helps to save storage space, but also tremendously contributes to the computational efficiency of the training algorithm.

To define 30 distinct features characterizing the time series, the one-hour time response comprising 72,000 data points is divided into ten equal segments. Table 3 shows the selected 30 features corresponding to ten segments. It can be observed that the selected features are non-dimensional. For example, *µ_i_*, *S_i_*, and *K_i_* denote the mean, skewness, and kurtosis of the *i*-th segment, respectively (*i* =1, 2, ……, 10). The first ten features were non-dimensionalized because having a mean value feature with nondimensional features (i.e., *K* and *S*) will be incompatible for any ML algorithm.

**Figure 3:**
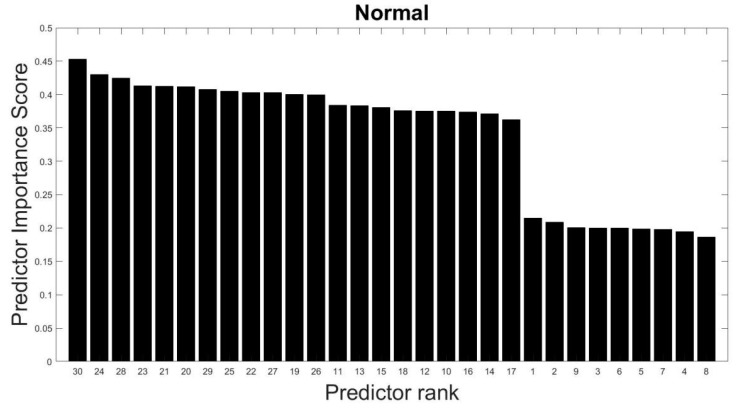
Predictor importance scores of 30 features for distinguishing between normal and anomalous time series.

**Figure 4:**
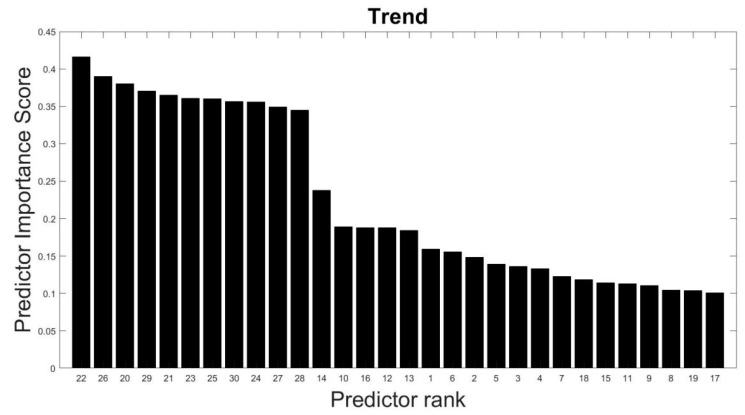
Predictor importance scores of 30 features for distinguishing between trend and other anomalous time series.

The selected features are tested using MRMR algorithm (as explained in Section 2.3) to confirm their suitability toward the prediction of the target variable (anomalous class). It is found that these features (predictors) have high predictor scores. For example, Figure 3 shows the importance of the 30 features (or predictors) to distinguish between normal and six other anomalies as evaluated from the training data. The importance of the features (or predictors) is measured by a predictor importance score varying from 0 to 1, where 0 is least important and 1 is most important. Feature numbers are depicted on the x-axis in descending order of their importance. As shown in Figure 3, it can be found that 21 features starting with feature #30 have predictor importance scores lying between 0.35 and 0.45. It indicates that these 21 features roughly hold equal importance in predicting the outcome, i.e., whether the time series is normal or an anomaly. The remaining nine features have predictor importance scores lying between 0.15 and 0.25. It indicates that these features are relatively less important than the aforementioned 21 features. It was found that on dropping the nine features with low predictor importance scores, the classification performance of the model is adversely affected. It indicates that all the features should be retained to achieve optimal classification performance. Similarly, high predictor scores were found for the representative features corresponding to other models. Figure 4 shows the importance of the 30 predictors used to distinguish between trend and not trend, i.e., other anomalous time series. For brevity, predictor importance scores only correspond to the models distinguishing normal and not normal, and trend and not trend are illustrated here.

## 4. Simplified Anomaly Detection Approach

**Figure 5:**
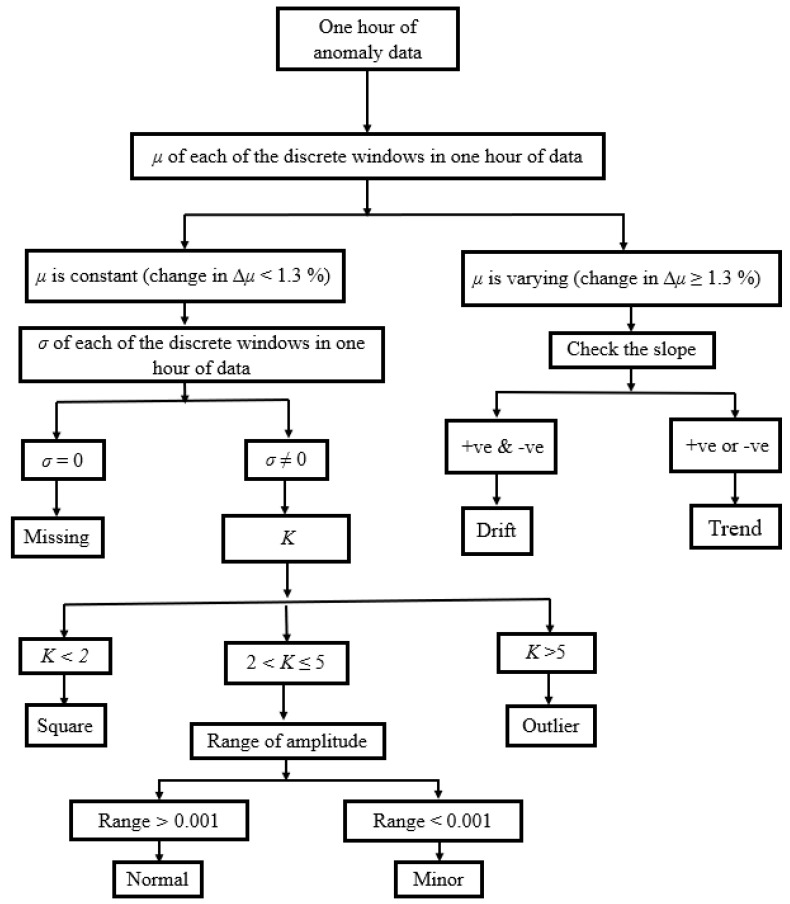
The flowchart of a simplified approach.

**Figure 6:**
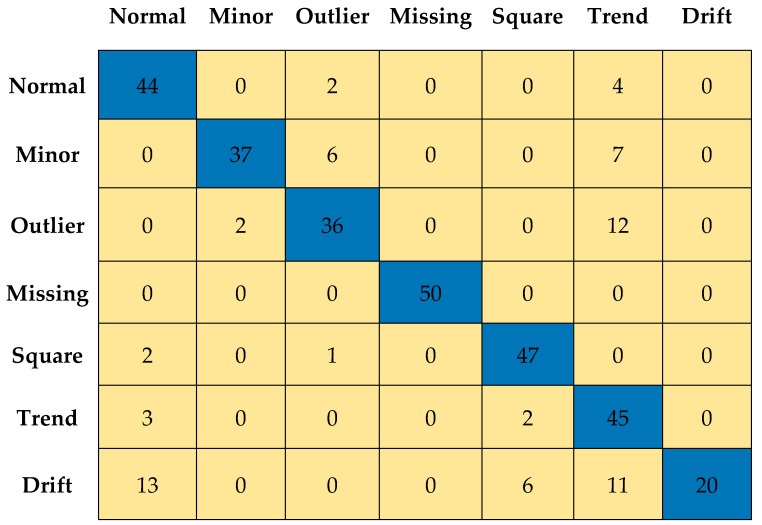
Confusion matrix of the anomaly data.

**Figure 7:**
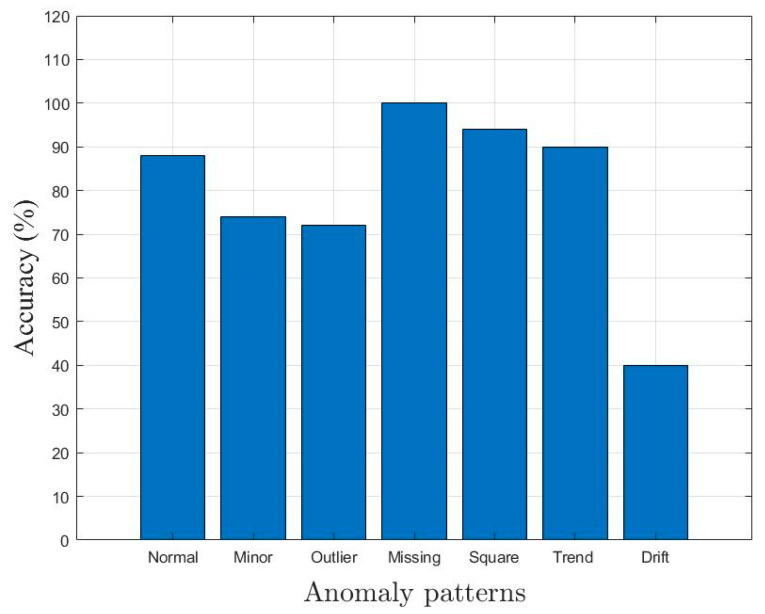
Accuracy of the simplified approach for all anomaly patterns.

In this section, the simplified statistical parameters, such as *µ*, *σ*, and *K* of time-series data, are used to classify the different anomaly patterns. Assuming *y(t)* is the measured vibration data of the bridge. *y*(*t*) is divided into several discrete segments, and the following simplified decision tree, as shown in Figure 5, is adopted to detect the anomalies. It was observed that the square anomaly is sub-Gaussian since its *K* value is less than 2, and the outlier anomaly is super-Gaussian due to its high *K* value, which exceeds 5. Therefore, the square and outlier anomalies were classified based on their kurtosis value (e.g., *K* of square anomaly < 2 and *K* of outlier anomaly > 5). However, the normal and minor anomalies have the same *K* value that ranges between 2 and 5. To classify normal and minor anomalies, the range of amplitude was used as an indicator. In order to evaluate the classification performance of the decision tree-based approach, 50 time series corresponding to each class (1 normal and 6 anomalies) were used. Figure 6 shows the confusion matrix obtained after applying the decision tree-based approach to the randomly selected 50 time series. The accuracy of classification performance is graphically illustrated in Figure 7. It can be observed that only missing anomalies were successfully detected using the simplified method. However, the data containing drift, minor, and outlier result in poor identification accuracy, which motivated to develop an ML-based anomaly detection method.

## 5. The Proposed ML-Based Recursive Binary Decision Tree for Anomaly Detection

In this section, a novel approach for multiclass time-series classification using the combination of decision tree and ML is proposed. It identifies one class at a time. First, the data is classified as normal and not normal using an ML-based classifier. Further, the not-normal data is classified as trend and not trend. A similar approach is implemented for classifying square, missing, minor, drift, and outlier. Eventually, all seven classes are identified, accomplishing a multiclass time-series classification. This process of successively classifying one class at a time is called a binary decision tree. The entire data is divided into training (70%), validation (20%), and testing datasets (10%) for training ML classifiers, fine tuning the classification performance of the ML classifiers and evaluating the classification accuracy of the ML classifiers, respectively. Table 4 shows the number of time series belonging to different classes in the training, validation, and testing dataset.

**Figure 8:**
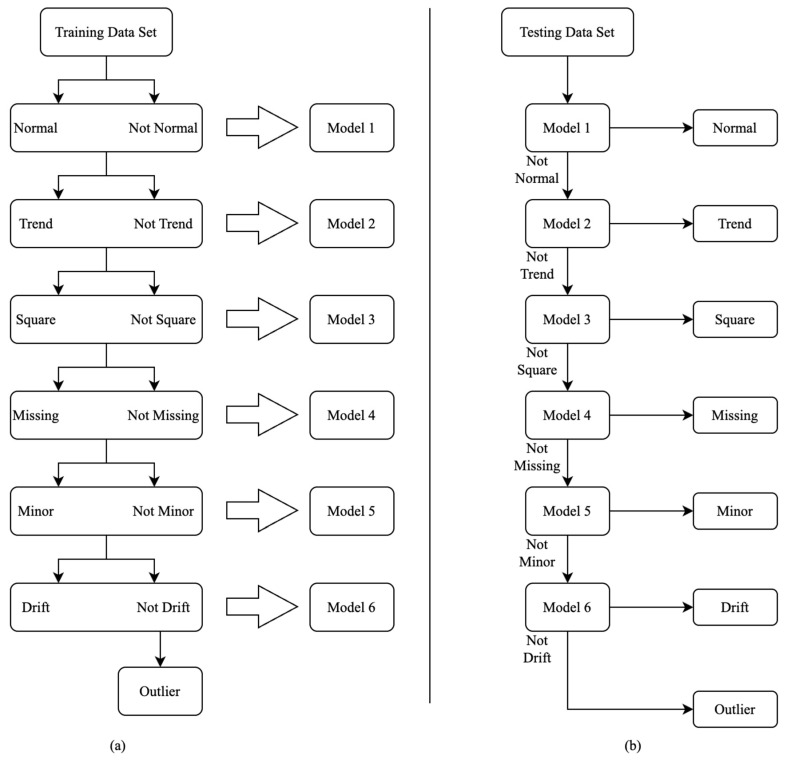
Schematic representation of the training (**a**) and testing (**b**) methodology for Models 1–6.

The first step is to train a set of models that constitute a binary decision tree to accomplish this classification. For that, all the time series belonging to the training set are labeled as normal and not normal (i.e., trend, square, missing, minor, drift, and outlier). Then, a supervised ML model called Model 1 is trained to perform a categorical classification of time series (normal and not normal) based on 30 features defined to characterize each time series. To further classify not normal, another supervised ML model is trained to distinguish between trend and not Trend (for classes that are square, missing, minor, drift, and outlier), which is named as Model 2. Model 2 is trained using all the time series belonging to the not normal set. Note that normal time series are not used while training Model 2, because they are supposedly classified by Model 1. A similar approach is adopted to classify the time series belonging to the classes—square (Model 3), missing (Model 4), minor (Model 5), drift (Model 6), and outlier in the same order, respectively. This order is the same as the numerical descending order of different time series present in the training, validation, and testing data set. Not adhering to this order will adversely affect the classification performance of the minority classes such as drift and outlier. Here, ensemble bagged trees or random forest classifier is used to train all the models constituting the binary decision tree. Figure 8a schematically represents the process of generating these models (i.e., training), and Figure 8b schematically represents the framework of the binary decision tree for accomplishing multiclass time-series classification (i.e., testing).

The cascade of Models 1 to 6—Level 0 is illustrated as follows. Consider, Model 1 of Level 0. This model may classify some normal as not normal (false negative) and some not normal as normal (false positive). Other models of Level 0 are also prone to this kind of problem. This problem is addressed by invoking the models of the binary decision tree recursively (i.e., augmenting the associated training data set each time) until satisfactory improvement in classification performance is achieved. The classification performance of Level 0 is measured on the validation data set, and a confusion matrix is generated. Analyzing the false positives and false negatives from the confusion matrix, the original training dataset associated with Model 1 is appropriately synthetically augmented using SMOTE (as explained in Section 2.2) to enhance the training process of Model 1. Based on the synthetically augmented dataset, Model 1 is redefined (other models are not redefined). The cascade of the redefined Model 1 and other Models (from 2 to 6) is called Level 1. Thus, Level 1 achieves an optimal classification performance for the normal class as affected by the synthetic augmentation of the training data set associated with Model 1.

To achieve optimal classification performance for the trend class, the classification performance of Level 1 is measured on the validation dataset, and a confusion matrix is generated. Analyzing the false positives and false negatives from the confusion matrix, the original training dataset associated with Model 2 is appropriately synthetically augmented using SMOTE to better train Model 2. Thus, Model 2 is redefined. Call the cascade of redefined Model 1, redefined Model 2, and other models (from 3 to 6)—Level 2. It may be noted that the original training data set associated with Model 2 only contained not normal (i.e., trend, square, missing, minor, drift, and outlier). However, the synthetically augmented training data set corresponding to redefined Model 2 may contain normal time series to identify normal wrongly classified as not normal by redefined Model 1 (no model can be 100% perfect). Thus, optimal classification performance is achieved for normal and trend time series. A similar approach is adopted until satisfactory classification performance is achieved for all the classes except outlier. All of not drift is not outlier. The presence of other classes (normal, trend, square, missing, minor, and drift) can be expected in not drift. Thus, it arises a need to define a new model called Model 7 to classify outlier from not drift. The training data set for Model 7 is engineered by analyzing the demographics of not drift after applying redefined models (1 to 6) on the validation data set. Call the cascade of all the redefined models (from 1 to 6) and Model 7—Level 7.

**Figure 9:**
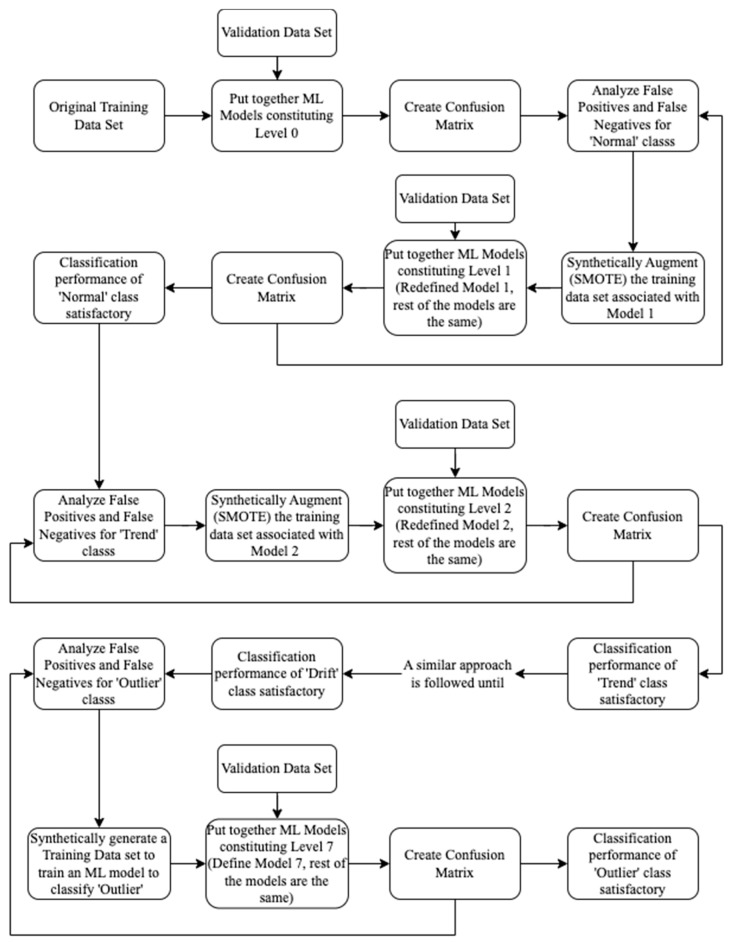
Schematic representation of the recursive decision tree.

In this section, ML models (1 to 6) constituting the binary decision tree are described. Subsequently, an approach to improve each of those models is presented wherein these models (1 to 6) are called recursively (i.e., making improvements each time) and tested on the validation data set. Thus, all the models are redefined to better perform the multiclass time-series classification. The creation of each redefined model leads to the creation of a new level (a cascade of 6 models, which undertakes multiclass classification), and there is one more level for improving the classification performance of the outlier class. The ML models employed in successive eight steps (or levels) are depicted in Table 5. Figure 9 illustrates the recursive approach of redefining ML models to achieve a better classification performance.

Following is the summarization of all the steps from data preparation to the development of the ML-based anomaly detection framework:

#1 Calculated a set of 30 characteristic features to represent each one-hour-long time series (see Section 3.2 and Table 3);
#2 Performed predictor importance or information value calculations to test the suitability of defined characteristic features toward the prediction of the target variable (anomalous class) using the MRMR algorithm (see Section 3.2, Figure 3 and Figure 4);
#3 Developed a simplified anomaly detection or classification framework using statistical parameters such as *µ*, *σ*, and *K* of time-series data (see Section 4 and Figure 5);
#4 Developed an ML-based binary decision tree framework using random forest classifiers (see Section 5 and Figure 8);
#5 Improved classification performance using an ML-based recursive binary decision tree and SMOTE (see Section 5 and Figure 9).

## 6. Results

### 6.1. Anomaly Detection Using Traditional Unsupervised ML

**Figure 10:**
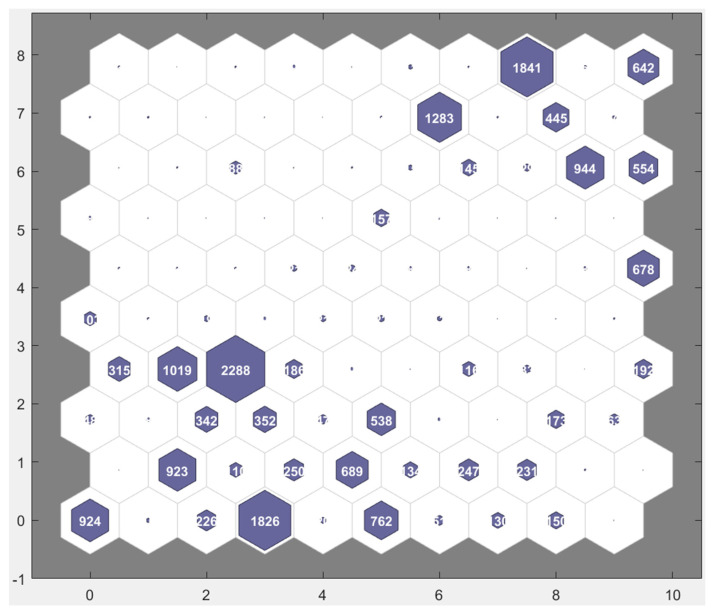
10 × 10 network of neurons forming an SOM of the training data.

A self-organizing map (SOM) is a data-clustering technique based on batch unsupervised weight/bias training. It trains a network with weight and bias learning rules with batch updates [28]. It topologically organizes the data by reducing its dimensionality. A 2D SOM represents data with ‘*n*’ features (*n* dimensions) on a 2D plane. This technique draws inspiration from the sensory and motor-mapping mechanisms found in mammal brains [28,29]. Here, 2D 10 × 10 SOM is used to determine the 30 distinct features that were described in Section 2.2, and render clear-cut boundaries between the seven classes (normal, trend, square, missing, minor, drift, outlier). Figure 10 shows the SOM of the training data generated using predefined features, where 100 neurons are arranged on a topological diagram depicting how many of the training data points (time series) are associated with each neuron (cluster centers). Visualizing the 2D map of cluster centers can help to develop a strategy for performing multiclass classification. It is evident that seven cluster centers (with clear-cut boundaries) corresponding to seven classes (normal, trend, square, missing, minor, drift, outlier) did not emerge. This makes it challenging to accomplish multiclass classification in a single step with the chosen set of 30 features. It may be possible to solve this problem better using higher-dimensional SOM and a better clustering algorithm. However, in this work, a supervised algorithm is developed that performed this classification in multiple steps, notwithstanding these challenges.

### 6.2. Anomaly Detection Using the Proposed Methodology

The implementation of the recursive decision-tree model is performed in eight consecutive steps (Level 0 to Level 7). In all, 70% of the data is used for training the ML models, and the remaining 30% to validate and test the trained machine-learning models. Level 0 is a binary decision tree made of ML models capable of performing multiclass time-series classification, as shown in Figure 8b and Table 5. Table 6 describes the data set used to train Model 1 to distinguish between normal and not normal, and Model 2 to distinguish between trend and not trend. Table 7 describes the data set used to train Model 3 to distinguish between square and not square, and Model 4 to distinguish between missing and not missing. Table 8 describes the data set used to train Model 5 to distinguish between minor and not minor, and Model 6 to distinguish between drift and not drift.

The classification performance of Level 0 is evaluated on the validation data set using the confusion matrix and performance metrics (i.e., F1 score, accuracy, recall, and precision). Analyzing the false positives and false negatives, the training data set associated with Model 1 is synthetically augmented using SMOTE to minimize the misclassifications of the normal data. This process is performed recursively to improve the classification performance of the normal data until a satisfactory classification performance for the normal data is obtained. Thus, Model 1 is redefined after synthetically augmenting the training data set associated with Model 1 (see Level 1 in Table 5), using a trial-and-error approach. Synthetic augmentation of the training data set in proportions other than that have been adopted may be suitable. However, the classification performance of the other classes should not be adversely affected. A similar approach is adopted to enhance the classification performance of the remaining classes, i.e., trend (redefined Model 2 for Level 2), square (redefined Model 3 for Level 3), missing (redefined Model 4 for Level 4), minor (redefined Model 5 for Level 5), drift (redefined Model 6 for Level 6), and outlier (Model 7 for Level 7), as shown in Table 5. Table 9 depicts the original data set and synthetically augmented data set used to train redefined Model 1 to distinguish between normal and not normal, and redefined Model 2 to distinguish between trend and not trend. Table 10 depicts the original and synthetically augmented data set used to train redefined Model 3 to distinguish between square and not square, and redefined Model 4 to distinguish between missing and not missing. Table 11 depicts the original and synthetically augmented data set used to train redefined Model 5 to distinguish between minor and not minor, and redefined Model 6 to distinguish between drift and not drift. Table 12 depicts the synthetically engineered data set used to train Model 7 to classify outlier and not outlier. It is important to follow the numerical descending order of different time series present in the training, validation, and testing data set. Not adhering to this order will adversely affect the classification performance of the minority classes.

**Figure 11:**
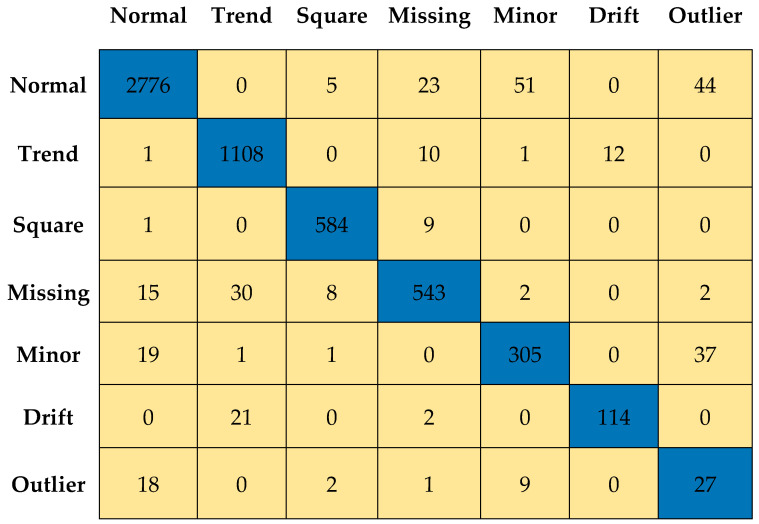
Confusion matrix for Level 0 using validation dataset.

**Figure 12:**
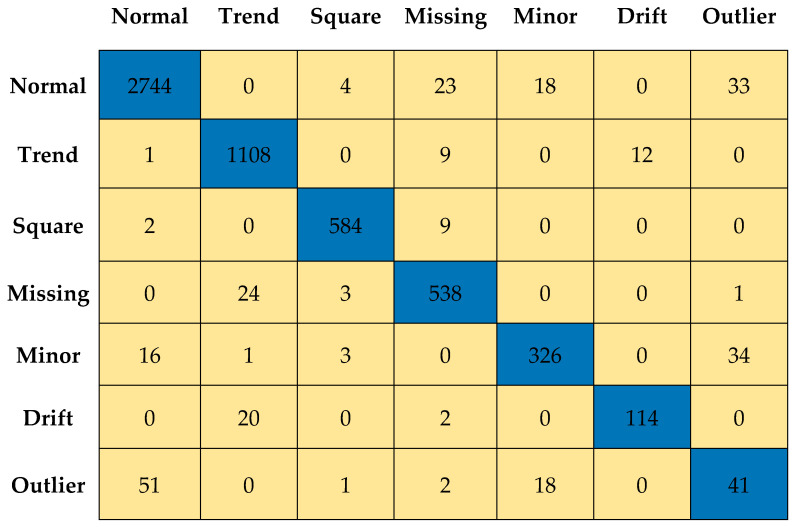
Confusion matrix for Level 7 using validation dataset.

**Figure 13:**
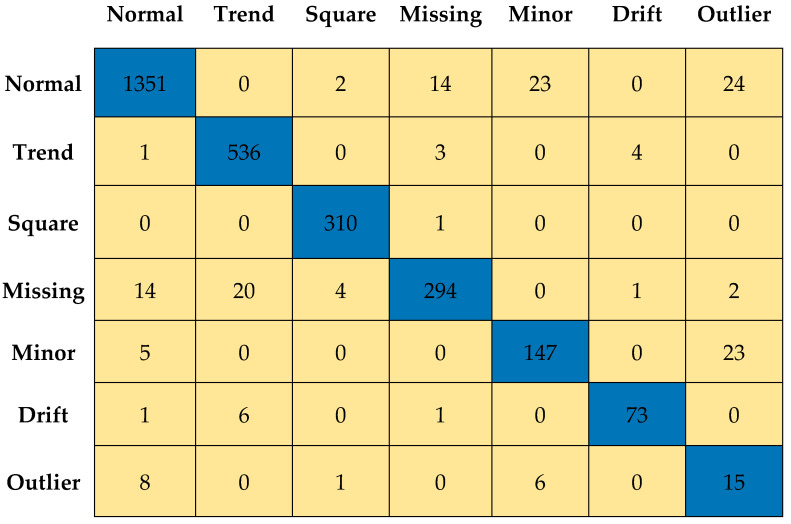
Confusion matrix for Level 0 using test dataset.

**Figure 14:**
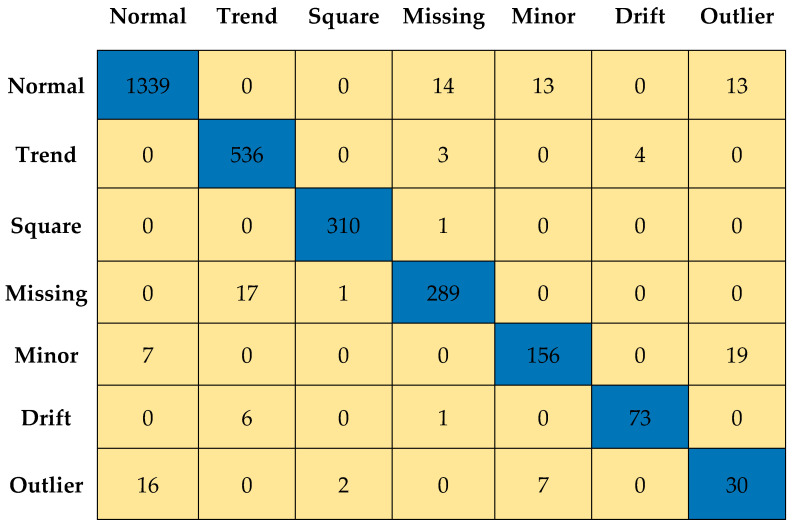
Confusion matrix for Level 7 using test data set.

**Figure 15:**
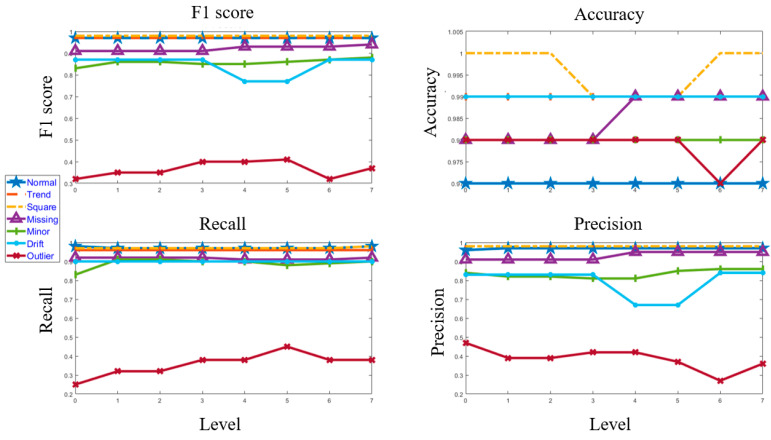
Evolution of the performance matrices from Level 0 to Level 7 for the validation dataset.

**Figure 16:**
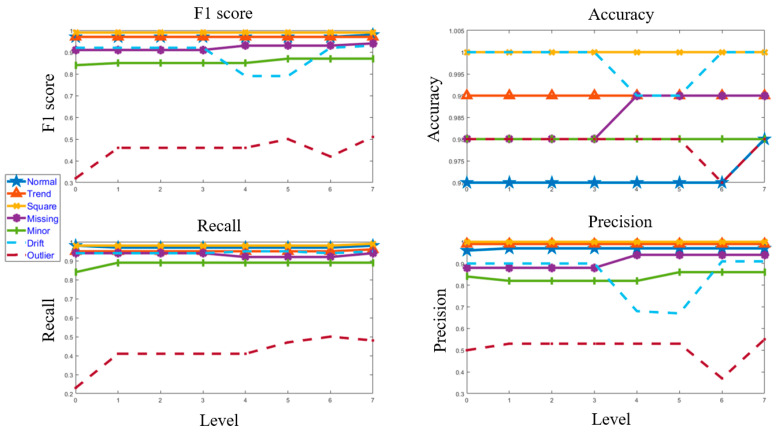
Evolution of the performance matrices from Level 0 to Level 7 for the test dataset.

Figure 11 and Figure 12 depict the confusion matrices for Level 0 (first level) and Level 7 (last level) evaluated on the validation data set. Figure 13 and Figure 14 depict the confusion matrices for Level 0 (first level) and Level 7 (last level) evaluated on the test data set. Quantitatively, the classification performance is measured using performance metrics—F1 score, accuracy, recall, and precision. The evolution of all the performance metrics is illustrated in Figure 15 and Figure 16 for validation and testing, respectively.

For the test dataset in Level 7 (after Model 7 is used), the classes that are classified as not outliers are ignored and not counted towards the true positive, true negative, false positive, or false negative for any of the seven classes; as the number of time series ignored was 32 (18 normal, 3 trends, 3 square, 5 missing, 1 drift, 2 outliers), a relatively small number in percentage terms for respective classes. The same is followed for the validation data set (in Level 7 after Model 7 is used).

## 7. Conclusions

In this paper, a recursive binary tree approach is proposed for multiclass anomaly detection. In the first level, a binary decision tree containing six ML models is developed to classify and predict different anomalies in data collected from a real-life bridge. Subsequently, all the ML models are redefined to minimize false positives and false negatives of normal, trend, square, missing, minor, drift, and outlier. The process of redefining the ML models employed SMOTE to synthetically augment the associated training datasets and obtain classification performance measured in terms of F1 score as 98%, 97%, 99%, 94%, 87%, 93%, and 51% for normal, trend, square, missing, minor, drift, and outlier, respectively. The accuracy of this classification model is 98%, 99%, 100%, 99%, 98%, 100%, and 98% or normal, trend, square, missing, minor, drift, and outlier, respectively (these metrics are calculated with precision up to the second decimal place). Moreover, the need for synthetic augmentation and multiple levels may be eliminated, contributing to the computational efficiency of the classification algorithm. In future, a similar recursive binary decision tree framework could be developed to solve unsupervised machine learning problems focusing on the maximization of predictability scores as against the maximization of performance metrics in supervised ML problems.

## Figures and Tables

**Table 1:** Description of the anomalies.

Class	Description
**Normal**	Time-domain response is symmetrical, whereas frequency-domain response has several resonance peaks.
**Trend**	An evident trend can be found in the time-domain response, whereas a distinctive peak value can be found in the frequency-domain response.
**Square**	The time-domain response resembles a square wave.
**Missing**	Most or all of the time-domain response is missing.
**Minor**	Relative to normal class data, the amplitude is very small in the time-domain response.
**Drift**	The time-domain response is non-stationary with random drift or time-domain response diverges with time.
**Outlier**	One or more outliers (i.e., high spikes) appear in the time-domain response.

**Table 2:** The number of different anomalies in the dataset.

Anomaly	Quantity
Normal	13,575
Trend	5778
Square	2996
Missing	2942
Minor	1775
Drift	679
Outlier	527

**Table 3:** Mathematical description of all the 30 distinct features.

Feature #	Feature Description	Feature #	Feature Description	Feature #	Feature Description
1	(μ_2_ − μ_1_)/μ₁	11	S₁	21	K₁
2	(μ_3_ − μ_2_)/μ₂	12	S₂	22	K₂
3	(μ_4_ − μ_3_)/μ₃	13	S₃	23	K₃
4	(μ_5_ − μ_4_)/μ₄	14	S₄	24	K₄
5	(μ_6_ − μ_5_)/μ₅	15	S₅	25	K₅
6	(μ_7_ − μ_6_)/μ₆	16	S₆	26	K₆
7	(μ_8_ − μ_7_)/μ₇	17	S₇	27	K₇
8	(μ_9_ − μ_8_)/μ₈	18	S₈	28	K₈
9	(μ_10_ − μ_9_)/μ₉	19	S₉	29	K₉
10	(max − min)/μ	20	S_10_	30	K_10_

**Table 4:** Description of training, validation, and testing dataset.

Class	Training	Validation	Testing
Normal	9365	2830	1380
Trend	4056	1160	562
Square	2079	600	317
Missing	2041	588	313
Minor	1231	368	176
Drift	475	126	78
Outlier	353	110	64

**Table 5:** Depiction of the ML models employed in 8 steps (or levels), ensuring successive improvement in the performance of multiclass classification.

Level 0	Level 1	Level 2	Level 3	Level 4	Level 5	Level 6	Level 7
Model 1	Redefined Model 1	Redefined Model 1	Redefined Model 1	Redefined Model 1	Redefined Model 1	Redefined Model 1	Redefined Model 1
Model 2	Model 2	Redefined Model 2	Redefined Model 2	Redefined Model 2	Redefined Model 2	Redefined Model 2	Redefined Model 2
Model 3	Model 3	Model 3	Redefined Model 3	Redefined Model 3	Redefined Model 3	Redefined Model 3	Redefined Model 3
Model 4	Model 4	Model 4	Model 4	Redefined Model 4	Redefined Model 4	Redefined Model 4	Redefined Model 4
Model 5	Model 5	Model 5	Model 5	Model 5	Redefined Model 5	Redefined Model 5	Redefined Model 5
Model 6	Model 6	Model 6	Model 6	Model 6	Model 6	Redefined Model 6	Redefined Model 6
							Model 7

**Table 6:** Description of training data sets for Model 1 and Model 2.

Identifying Normal			Identifying Trend		
	Class	Model 1		Class	Model 2
		Training Set			Training Set
**Type-1** **Normal**	**Normal**	9365	**Type-1 Trend**	Trend	4056
			
**Type-2** **Not Normal**	**Trend**	4056	**Type-2** **Not Trend**	Normal	0
			
	**Square**	2079		Square	2079
			
	**Missing**	2041		Missing	2041
			
	**Minor**	1231		Minor	1231
			
	**Drift**	475		Drift	475
			
	**Outlier**	353		Outlier	353
			

**Table 7:** Description of training data sets for Model 3 and Model 4.

Identifying Square			Identifying Missing		
	Class	Model 3		Class	Model 4
		Training Set			Training Set
**Type-1 Square**	**Square**	2079	**Type-1 Missing**	**Missing**	2041
			
**Type-2** **Not Square**	**Normal**	0	**Type-2** **Not Missing**	**Normal**	0
			
	**Trend**	0		**Trend**	0
			
	**Missing**	2041		**Square**	0
			
	**Minor**	1231		**Minor**	1231
			
	**Drift**	475		**Drift**	475
			
	**Outlier**	353		**Outlier**	353
			

**Table 8:** Description of training data sets for Model 5 and Model 6.

Identifying Minor			Identifying Drift		
	Class	Model 5		Class	Model 6
		Training Set			Training Set
**Type-1 Minor**	**Minor**	1231	**Type-1 Drift**	**Drift**	475
			
**Type-2** **Not Minor**	**Normal**	0	**Type-2** **Not Drift**	**Normal**	0
			
	**Trend**	0		**Trend**	0
			
	**Square**	0		**Square**	0
			
	**Missing**	0		**Missing**	0
			
	**Drift**	475		**Minor**	0
			
	**Outlier**	353		**Outlier**	353
			

**Table 9:** Description of training data sets used for the redefined Models 1 and 2.

Identifying Normal				Identifying Trend			
	Class	Model 1	Redefined Model 1		Class	Model 2	Redefined Model 2
		Original Training Set	After Synthetic Augmentation			Original Training Set	After Synthetic Augmentation
**Type-1** **Normal**	**Normal**	9365	13,111	**Type-1** **Trend**	**Trend**	4056	4056
			↑ 40%				↑ 0%
**Type-2 Not Normal**	**Trend**	4056	4056	**Type-2 Not Trend**	**Normal**	0	0
			↑ 0%				↑ 0%
	**Square**	2079	2079		**Square**	2079	2079
			↑ 0%				↑ 0%
	**Missing**	2041	5103		**Missing**	2041	2041
			↑ 150%				↑ 0%
	**Minor**	1231	3078		**Minor**	1231	1231
			↑ 150%				↑ 0%
	**Drift**	475	475		**Drift**	475	475
			↑ 0%				↑ 0%
	**Outlier**	353	1765		**Outlier**	353	353
			↑ 400%				↑ 0%

**Table 10:** Description of training data sets used for the redefined Models 3 and 4.

Identifying Square				Identifying Missing			
	Class	Model 3	Redefined Model 3		Class	Model 4	Redefined Model 4
		Original Training Set	After Synthetic Augmentation			Original Training Set	After Synthetic Augmentation
**Type-1 Square**	**Square**	2079	2079	**Type-1** **Missing**	**Missing**	2041	2041
			↑ 0%				↑ 0%
**Type-2 Not Square**	**Normal**	0	0	**Type-2 Not Missing**	**Normal**	0	2000
			↑ 0%				↑ 21.4%
	**Trend**	0	0		**Trend**	0	2000
			↑ 0%				↑ 49.3%
	**Missing**	2041	2041		**Square**	0	0
			↑ 0%				↑ 0%
	**Minor**	1231	1231		**Minor**	1231	1231
			↑ 0%				↑ 0%
	**Drift**	475	475		**Drift**	475	475
			↑ 0%				↑ 0%
	**Outlier**	353	353		**Outlier**	353	1412
			↑ 0%				↑ 300%

**Table 11:** Description of training data sets used for redefined Models 5 and 6.

Identifying Minor				Identifying Drift			
	Class	Model 5	Redefined Model 5		Class	Model 6	Redefined Model 6
		Original Training Set	After Synthetic Augmentation			Original Training Set	After Synthetic Augmentation
**Type-1 Minor**	**Minor**	1231	4924	**Type-1** **Drift**	**Drift**	475	2375
			↑ 300%				↑ 400%
**Type-2 Not Minor**	**Normal**	0	2500	**Type-2 Not Drift**	**Normal**	0	0
			↑ 26.7%				↑ 0%
	**Trend**	0	0		**Trend**	0	2000
			↑ 0%				↑ 49.3%
	**Square**	0	0		**Square**	0	0
			↑ 0%				↑ 0%
	**Missing**	0	0		**Missing**	0	0
			↑ 0%				↑ 0%
	**Drift**	475	475		**Minor**	0	0
			↑ 0%				↑ 0%
	**Outlier**	353	1765		**Outlier**	353	1765
			↑ 400%				↑ 400%

**Table 12:** Description of training datasets used for redefined Model 7.

Identifying Outlier			
	Class		Model 7
		No Model	After Synthetic Augmentation
**Type-1 Outlier**	**Outlier**	353	1765
			↑ 400%
**Type-2 Not Outlier**	**Normal**	0	300
			↑ 3.2%
	**Trend**	0	100
			↑ 2.5%
	**Square**	0	100
			↑ 4.8%
	**Missing**	0	50
			↑ 2.4%
	**Minor**	0	150
			↑ 12.2%
	**Drift**	0	50
			↑ 10.5%

## Data Availability

The source of the selected database is cited in the manuscript [22], and acknowledged in the following subheading.
